# **In-silico** gene essentiality analysis of polyamine biosynthesis reveals APRT as a potential target in cancer

**DOI:** 10.1038/s41598-017-14067-8

**Published:** 2017-10-30

**Authors:** Jon Pey, Edurne San José-Eneriz, María Carmen Ochoa, Iñigo Apaolaza, Pedro de Atauri, Angel Rubio, Xabier Cendoya, Estíbaliz Miranda, Leire Garate, Marta Cascante, Arkaitz Carracedo, Xabier Agirre, Felipe Prosper, Francisco J. Planes

**Affiliations:** 1 Bioinformatics Group, CEIT and TECNUN, University of Navarra, San Sebastian, 20018 Spain; 2 Mathematics for Life (M4L), San Sebastian, 20018 Spain; 3 Hemato-Oncology Division, IDISNA, Centro de Investigación Médica Aplicada (CIMA), University of Navarra, Pamplona, 31008 Spain; 4 CIBERONC, Instituto de Salud Carlos III, Madrid, 28029 Spain; 5 Department of Biochemistry and Molecular Biology, University of Barcelona, Barcelona, 08028 Spain; 6 CIC bioGUNE, Bizkaia Technology Park, 801 Building, 48160 Derio, Spain; 7 Ikerbasque, Basque foundation for science, 48011 Bilbao, Spain; 8 Biochemistry and Molecular Biology Department, University of the Basque Country (UPV/EHU), P. O. Box 644, E-48080 Bilbao, Spain

## Abstract

Constraint-based modeling for genome-scale metabolic networks has emerged in the last years as a promising approach to elucidate drug targets in cancer. Beyond the canonical biosynthetic routes to produce biomass, it is of key importance to focus on metabolic routes that sustain the proliferative capacity through the regulation of other biological means in order to improve *in-silico* gene essentiality analyses. Polyamines are polycations with central roles in cancer cell proliferation, through the regulation of transcription and translation among other things, but are typically neglected in *in silico* cancer metabolic models. In this study, we analysed essential genes for the biosynthesis of polyamines. Our analysis corroborates the importance of previously known regulators of the pathway, such as Adenosylmethionine Decarboxylase 1 (AMD1) and uncovers novel enzymes predicted to be relevant for polyamine homeostasis. We focused on Adenine Phosphoribosyltransferase (APRT) and demonstrated the detrimental consequence of APRT gene silencing on different leukaemia cell lines. Our results highlight the importance of revisiting the metabolic models used for *in-silico* gene essentiality analyses in order to maximize the potential for drug target identification in cancer.

## Introduction

Constraint-based modeling (CBM) represents one of the most popular approaches within computational systems biology. It focuses on building, contextualizing and analysing genome-scale metabolic networks^1^. CBM integrates different types of mathematical and biophysical constraints, enabling prediction of metabolic phenotypes. In particular, CBM allows us to conduct gene essentiality analysis (GEA), which aims at determining (i) essential genes, defined as those genes which individual deletion is incompatible with organism/cell growth; and (ii) synthetic lethal genes, which refer to two (or more) non-essential genes which simultaneous deletion becomes incompatible with cellular proliferation.

CBM and GEA have been successfully applied in the field of microbiology^2^. Growth is here modelled as the flux of a physiological reaction, typically named biomass equation, which involves the metabolic requirements, in terms of building blocks and energy, to produce biomass. GEA is accomplished by computationally finding those genes whose individual (or simultaneous) deletion prevents the biomass reaction from being active. Essential (or synthetic lethal) genes are identified by obtaining the subset of reactions fully dependent on them and then confirming that the maximum biomass production becomes zero when these reactions are deleted from the metabolic model. Note here that enzymes catalyzing metabolic reactions are usually a gene product or, in the case of heterodimers or multimers, the junction of several gene products. The set of genes needed for a reaction to take place can be inferred based on the boolean rules typically included in genome-scale metabolic networks^3^.

Over the last few years, different studies have emerged to extend this same approach to the field of cancer research, exploiting the release of high-quality human genome-scale metabolic networks^3,4^ and extensive availability of transcriptomic and proteomic data. The first successful application of GEA in cancer was presented in Folger *et al*.^5^. They revealed that *haem oxygenase* is synthetically lethal with the tumor suppressor *fumarate hydratase*. This result was later experimentally validated, showing its relevance to treat leiomyomatosis and renal cell cancer^6^.

In order to further continue and extend this seminal work, much effort has been done in developing algorithms for contextualizing a reference human metabolic network for each particular scenario mainly based on gene expression data. In essence, these methods aim to select the consistent subset of reactions active for a given cancer sample. A non-exhaustive list of this type of methods includes: GIMME^7^, iMAT^8^, FastCore^9^ and others^10^. With these approaches, the construction of different cancer-specific metabolic networks is now possible.

However, less work has been directed to contextualize and enrich the biomass equation in cancer. The biomass equation allows us to simulate the biosynthesis of essential metabolites to produce new cells, a feature of particular relevance in cancer given the highly proliferative phenotype of malignant cells. The first biomass equation was released by Folger *et al*.^5^, which involves 42 essential metabolites in human tissues, including nucleotides, deoxynucleotides, amino acids and lipids. Despite its novelty at the time, this biomass definition is limited and requires further development. The process of cell growth and proliferation requires metabolic reactions that do not directly provide metabolites used for biomass, but rather molecules that will support the activation of signalling and regulatory programs. One remarkable example is 2-hydroxyglutarate, a metabolite arising from mutated IDH in a number of tumors^11^, which has been shown to modify the epigenetic profile in cancer. Other important oncometabolites are acetate^12^, succinate or fumarate^13^. These observations emphasize the need to consider additional cancer-specific metabolic phenotypes, which can be systematically impaired via gene essentiality analysis. Two recent studies have focused on this growing need, providing an extended biomass equation^14^ or metabolic requirements associated with highly migratory cancer cells^15^.

In this study we focused on the requirement of polyamines (spermidine, putrescine and spermine) for tumor cell proliferation^16,17^. Polyamines are oncometabolites that exert their action, at least in part, by regulating different cancer signalling pathways^18,19^ and are over-produced by cancer cells^20–22^. Inhibition of polyamine biosynthesis has been largely investigated as a potential anti-cancer strategy, leading to a number of promising targets and inhibitors^23,24^. However, polyamines are typically neglected in the biomass equation of existing *in-silico* cancer metabolic models and, therefore, a gene essentiality analysis for polyamine biosynthesis in cancer is still lacking. In this study, we focused on the identification of genetic perturbations that hamper polyamine production and propose Adenine Phosphoribosyltransferase (APRT) as a candidate target to inhibit the production of these biomolecules and, in turn, curb cancer cell growth.

## Results

### Gene essentiality analysis for polyamine biosynthesis in cancer

We used the generic cancer model presented in Folger *et al*.^5^, which was built based on the expression data of the NCI-60 human cancer cell line panel and the standard RPMI-1640 growth medium conditions used in these cell cultures. The biomass equation presented in Folger *et al*.^5^ was amended by including polyamines at similar levels as found in other reconstructions^25^ (see Methods section). Under this new scenario, we repeated the gene essentiality analysis as conducted in Folger *et al*.^5^. Table 1 summarizes the set of essential and synthetic lethal genes for the biosynthesis of polyamines arising from our computational study. Note that the results reported here were not initially obtained in Folger *et al*.^5^, as their role is intrinsically linked to the biosynthesis of polyamines. Given that polyamine biosynthesis plays a prominent role in cancer cells over healthy tissues^20–22^, we hypothesized that the set of essential and synthetic lethal genes shown in Table 1 would be tumor-specific.

**Table 1:** List of essential and synthetic lethal genes for the biosynthesis of polyamines obtained in our cancer study. This list was obtained after applying gene essentiality analysis to the *in-silico* cancer metabolic model presented in Folger *et al*.^5^, using an updated biomass equation that involves putrescine, spermidine and spermine in the amounts typically found in other reconstructions (see Methods section).

**Gene(s)**	**Enzyme(s)**	**Type**
AMD1	adenosylmethionine decarboxylase	Essential
MTAP	5′-methylthioadenosine phosphorylase	Essential
ODC1	ornithine decarboxylase	Essential
SRM	spermidine synthase	Essential
MAT1A & MAT2A	methionine adenosyltransferase	Synthetic
MAT1A & MAT2B	methionine adenosyltransferase	Synthetic
APRT & PNP	adenine phosphoribosyltransferase & purine-nucleoside phosphorylase	Synthetic
ARG1 & OAT	arginase & ornithine transaminase reversible	Synthetic

AMD1, MTAP, ODC1 and SRM are directly involved in the polyamines biosynthesis and their role in cancer has been previously studied^17^. Indeed, inhibition of ODC1 with difluoromethylornithine (DFMO) is suggested to reduce the risk of colorectal cancer^26^ and targeting AMD1 with SAM486A reduces prostate cancer growth with negligible systemic toxic effects^22^. MTAP deletion is frequently observed in cancer^27^, suggesting that additional pathways compensate the lack of this gene. In particular, in the light of the recently elucidated synthetic lethality of MTAP with PRMT5^28^, MTAP-deleted cells seem to rewire their metabolism to elicit an epigenetic switch that explains their selective advantage. Our computational analysis is not capable of predicting the side-benefits of depleting MTAP due to its specific nature on metabolic modeling.

MAT1A & MAT2A and MAT1A & MAT2B were revealed as synthetic lethal genes in our analysis. Interestingly, the metabolic model presented in Folger *et al*.^5^ does not take into account tissue-specific gene expression. Since MAT1A is predominantly produced in the liver^29^, our results would suggest that MAT2A and MAT2B are essential genes in other cancer tissues. MAT enzymes catabolize methionine and produce S-adenosylmethionine (SAM), the major methyl donor in the cell and the precursor of decarboxylated SAM for polyamine synthesis. The results are therefore coherent with the essential nature of enzymes that are required for the production of polyamine precursors. In fact, the inhibition of MAT2A has been proved to decrease cell growth in colon cancer^30^.

ARG1 and OAT are the only enzymes included in the metabolic model in Folger *et al*.^5^ capable of producing *ornithine*, key precursor of polyamines. In turn, the synthetic lethality scored in our model is consistent with the negative consequences of ornithine ablation on polyamine production. Indeed, experiments in yeast showed that their inhibition significantly decreases the level of polyamines^31^.

Strikingly, two genes with no apparent relationship to polyamine production were highlighted as synthetic lethal in our model, purine-nucleoside phosphorylase (PNP) and adenine phosphoribosyltransferase (APRT). On the one hand, the inhibition of PNP was previously identified as a promising therapeutic strategy in T-cell acute lymphoblastic leukemia (T-ALL) and cutaneous T-cell lymphoma (CTCL)^32^. This led to the development of different PNP inhibitors, such as forodesine, which has been tested in a number of clinical trials^33^. The cytotoxicity derived from PNP inhibition in these tumors is mediated by the accumulation of dGTP, which leads to the apoptosis of lymphocytes^34^. Interestingly, the association of PNP inhibition with the biosynthesis of polyamines has not been reported and requires further consideration. On the other hand, the role of APRT in cancer is poorly understood and constitutes an attractive target to be explored. It catalyzes the reaction that synthesizes AMP from adenine. Importantly, lack of APRT activity results in the accumulation of a toxic by-product of adenine, 2,8-dihydroxyadenine (DHA)^35^.

### Proof-of-concept of the relevance of APRT in cancer

**Figure 1:**
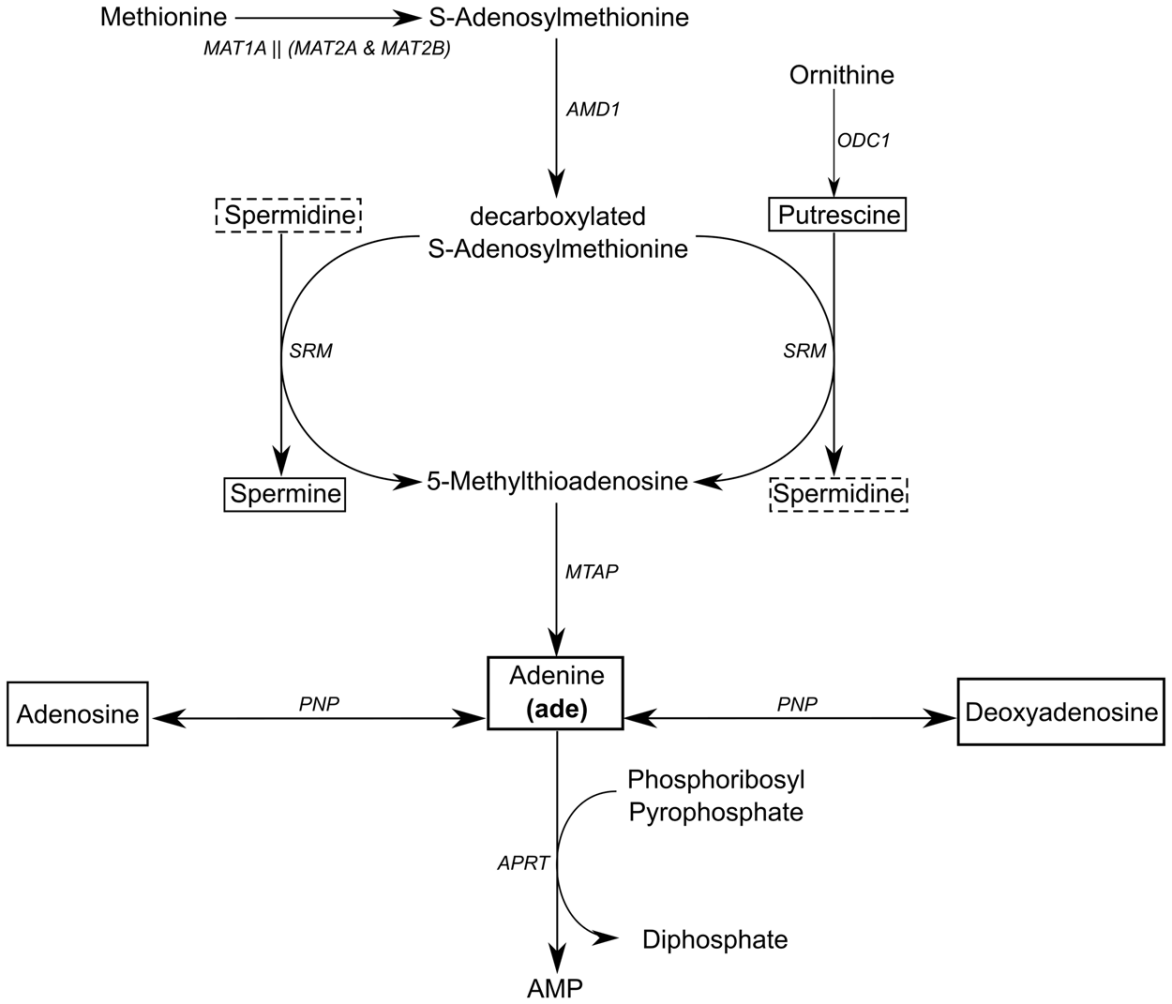
Genes and reactions involved in the biosynthesis of polyamines. *Putrescine* and *spermine* appear in an empty box, while *spermidine* in an empty dashed box. For clarity, *spermidine* is represented twice. Abbreviations: AMP: *adenosine monophosphate*.

Figure 1 shows a metabolic scheme of the enzymes involved in the production of polyamines in the study by Folger *et al*.^5^. Putrescine is the precursor for the formation of both spermidine and spermine; 5-methylthioadenosine (MTA) is a by-product in such biosynthetic pathway. There is only one enzyme consuming MTA, namely MTAP, which produces a molecule of adenine. It can be also observed that PNP and APRT consume adenine in order to produce adenosine/deoxyadenosine and AMP, respectively. In the cancer metabolic network presented in Folger *et al*.^5^, these reactions are the unique mechanisms consuming adenine.

Based on the above, the possible synthetic lethality of PNP and APRT arises as a consequence of the need to consume the generated adenine when spermidine and spermine are produced. We next proceeded to evaluate the synthetic lethality underlying APRT and PNP inhibition. In line with previous studies about PNP inhibition^36^, we focused on hematological malignancies, which additionally show a major dependence on polyamines than solid tumors^37^. In particular, B-ALL constitutes an interesting case where the synthetic lethality of PNP and APRT (if existing) could be relevant, since preliminary results about PNP inhibition show a significantly lower effect than in T-ALL^38^.

**Figure 2:**
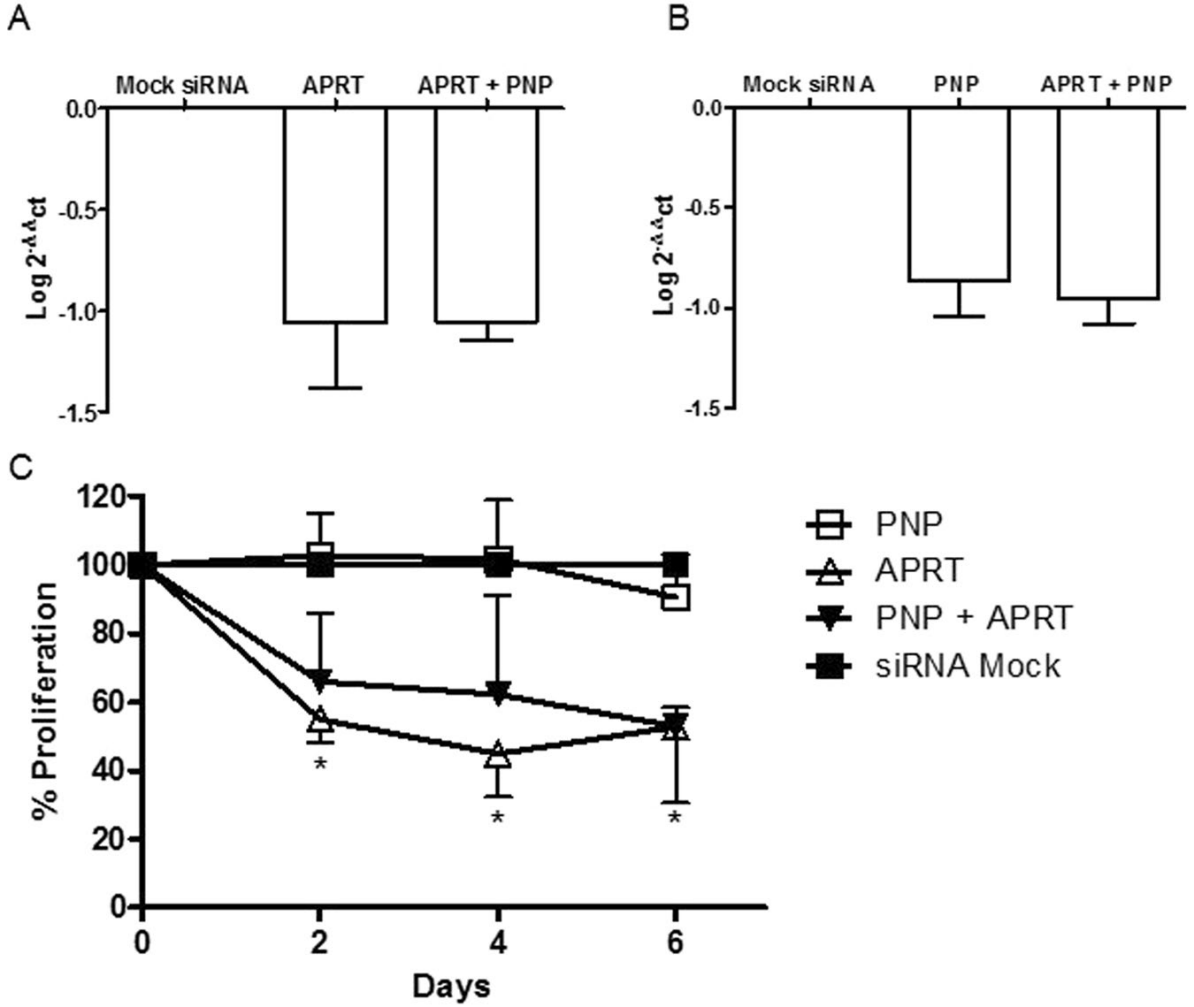
Gene silencing analysis of APRT and PNP in ALL derived CEMO-1 cell line. (**A** and **B**) mRNA expression of the APRT and PNP genes 48h after nucleofection with the siRNAs. Data are referred to GUS human gene and an experimental group nucleofected with mock siRNA. Data represent mean ± standard deviation of four pooled experiments with similar results. C) Proliferation of CEMO-1 cell line nucleofected with siRNAs targeted to the indicated genes was studied by MTS. *Indicate p-value < 0.001 in a repeated measures ANOVA test followed by a Bonferroni post-test between the PNP and APRT groups. Data represent mean ± standard deviation of four pooled experiments with similar results.

We conducted an *in vitro* gene silencing experiments in the B-ALL derived cell line CEMO-1. We measured cell proliferation over time in the following gene knockdown scenarios: i) control; ii) PNP knockdown; iii) APRT knockdown and iv) PNP and APRT knockdown. Results are summarized in Fig. 2, where the inhibition of APRT shows a relevant impact in decreasing CEMO-1 proliferation. On the other hand, the effect of PNP knockdown on proliferation, as well as its simultaneous knockdown with APRT, is non-significant compared to their respective controls. These results indicate that PNP and APRT are not synthetic lethal, while APRT is essential per se. Further details regarding the experimental protocols are included in the Methods section.

We then re-evaluated the function of PNP and APRT in order to explain the lack of experimental validation. A detailed analysis of the reactions associated to PNP shows that it can accept multiple substrates: adenine, guanine, hypoxanthine and uracil (Supplementary Table 1). Interestingly, while PNP accepts adenine as a substrate in prokaryotic cells, this is not the case in human cells^39^. Therefore, the degradation of adenine is incorrectly annotated to PNP, which explains the lack of synthetic lethality. In turn, in the absence of a compensatory PNP activity to metabolize adenine, our results would suggest that APRT is an essential gene in this setting. To confirm this, we amended the reactions annotated to PNP in the cancer metabolic model presented in Folger *et al*.^5^ and repeated the computational analysis carried out in the previous section, finding that APRT is now essential, in line with the reduction in cancer cell growth elicited by APRT silencing in Fig. 2. Table 2 shows the final set of essential and synthetic lethal genes resulting from our computational analysis once PNP was correctly annotated.

**Table 2:** List of essential and synthetic lethal genes for the biosynthesis of polyamines obtained in our cancer study once PNP annotation was corrected. With respect to Table 1, PNP and APRT are removed as synthetic lethal genes, while APRT now appears as essential gene.

Gene(s)	Enzyme(s)	Type
AMD1	adenosylmethionine decarboxylase	Essential
MTAP	5′-methylthioadenosine phosphorylase	Essential
ODC1	ornithine decarboxylase	Essential
SRM	spermidine synthase	Essential
APRT	adenine phosphoribosyltransferase	Essential
MAT1A & MAT2A	methionine adenosyltransferase	Synthetic
MAT1A & MAT2B	methionine adenosyltransferase	Synthetic
ARG1 & OAT	arginase & ornithine transaminase reversible	Synthetic

### Essentiality of APRT in acute leukemias via gene silencing

**Figure 3:**
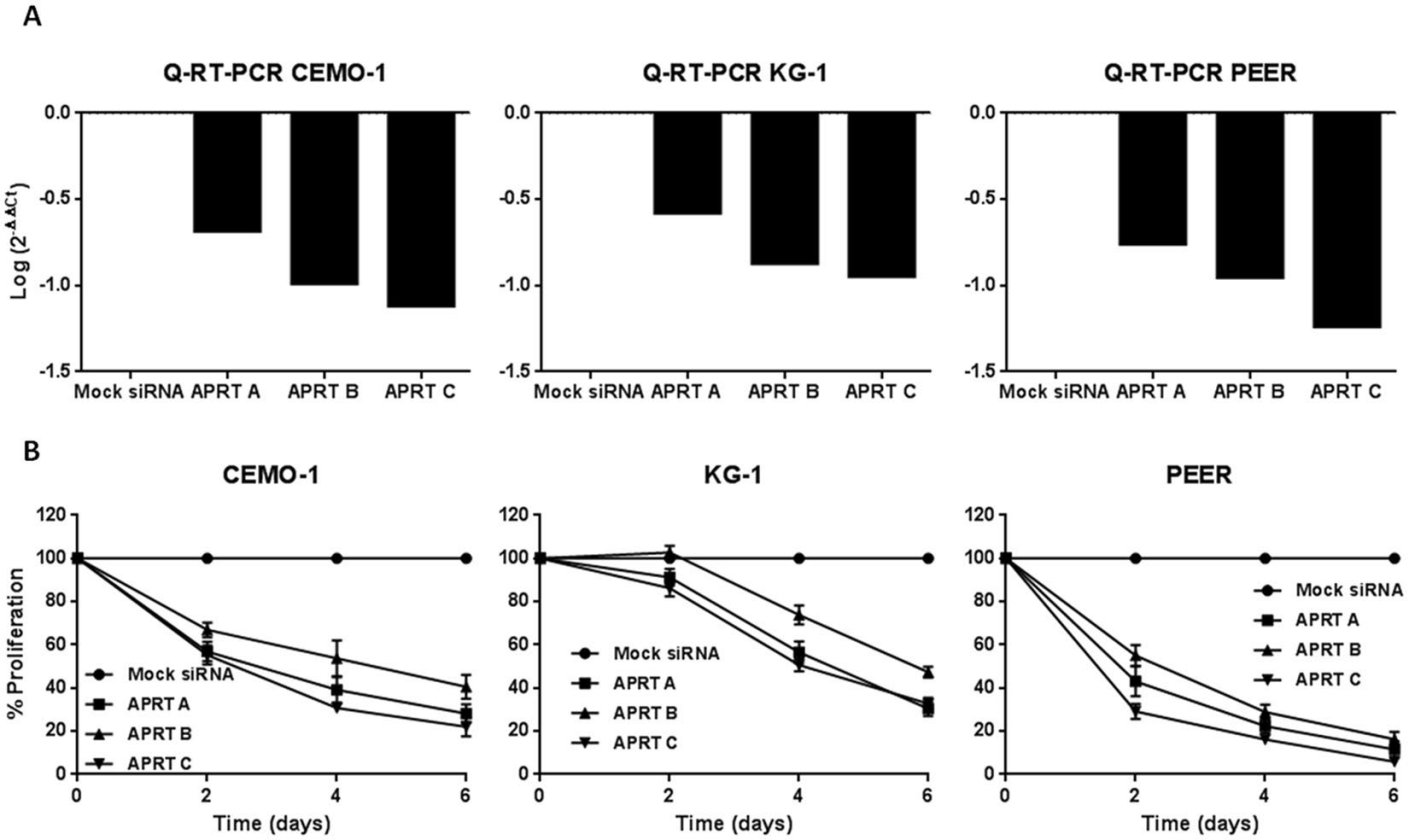
Gene silencing analysis of APRT in acute leukemias cell lines. (**A**) mRNA expression of *APRT* gene 48h after nucleofection with the siRNAs. Data are referred to GUS human gene and an experimental group nucleofected with Silencer Select Negative Control-1 siRNA (Mock siRNA). (**B**) Cell proliferation of CEMO-1, KG-1 and PEER cell lines nucleofected with APRT siRNAs studied by MTS. Data represent mean ± standard deviation of three different experiments.

In order to provide further insights into the nature of APRT as essential gene, we extended the analysis and conducted a more detailed *in vitro* silencing in several acute leukemia cell lines: CEMO-1, KG-1 (Acute myeloid leukemia (AML)), PEER, MOLT-4 (T-ALL), and MY (B-ALL). We used three siRNAs and each of them decreased *APRT* expression in all cell lines tested as detected by Q-RT-PCR (Fig. 3A, Supplementary Fig. 3a). Furthermore, cell proliferation was dramatically reduced after silencing of *APRT* expression in CEMO-1, KG-1, PEER cell lines (Fig. 3B), achieving reductions greater than 60% in all cases. Note that CEMO-1 cell line exhibits a similar result as the one found in Fig. 2. Regarding MOLT-4 and MY cell lines, we did not find any effect in cell proliferation, suggesting that APRT is not essential in all cell types (Supplementary Fig. 3b).

**Figure 4:**
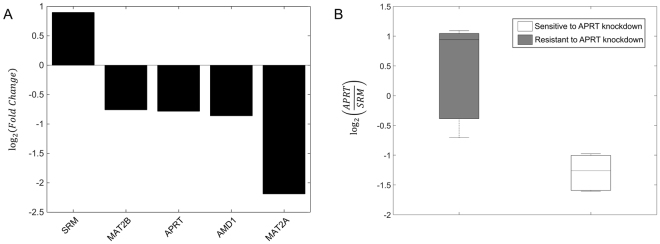
Gene expression analysis of cells sensitive and resistant to APRT knockdown for genes involved in the polyamines biosynthesis pathway. (**A**) Log (base 2) fold change estimates of differentially expressed genes (adj. p-value ≤ 0.05) involved in polyamines biosynthesis pathway. A gene with a positive fold change is upregulated in cells sensitive to APRT knockdown; (**B**) Boxplot of Log (base 2) APRT/SRM ratio in cells sensitive and resistant to APRT knockdown.

Our metabolic modeling assumes that cancer cells will benefit from maximal polyamine synthesis. Since APRT was not essential in all cell lines, we performed subsequent analyses in order to evaluate the molecular differences between cells sensitive and resistant to the silencing of the enzyme. Transcriptional analysis of these cells revealed changes in the abundance of mRNAs coding for polyamine biosynthetic enzymes, including MAT2A, MAT2B, AMD1, APRT and SRM (FDR ≤ 5%, see Methods section, Supplementary Fig. 4). Aside from SRM, the rest of the genes presented a lower expression in APRT-sensitive cell lines, as observed in Fig. 4A. These results suggest that the abundance of polyamine biosynthetic enzymes could determine the essentiality of APRT in cancer and confirm the association between the effect of APRT inhibition and polyamines.

## Discussion

The study of cellular metabolism in cancer research has been actively reawakened in the last years. Systems biology approaches, based on high-throughput “omics” data and genome-scale metabolic networks, are suitable for studying cancer metabolism at an unprecedented level of detail and may provide valuable insights for different relevant clinical questions. Here, we use CBM and gene essentiality analysis, a promising computational tool for identifying therapeutic targets in cancer, in order to identify novel strategies to disrupt polyamines production in cancer.

We first emphasized the need of refining constraint-based models in order to include tumor-specific phenotypes that can be targeted via gene essentiality analysis. Much effort has been done in developing algorithms for contextualizing a reference human metabolic network for a particular cancer type. However, improving biomass equations in cancer models has received less attention. The development of cancer-specific biomass equations may lead to the identification of novel specific targets for malignant cells. This is shown in our study, where APRT could be a cancer-specific target, given that cancer cells exhibit a higher dependence on polyamines over healthy cells^20–22^.

Continuing this research line will undoubtedly be an important task in the next years due to the urgent need of developing more effective and personalized cancer therapies. In our opinion, the development of algorithms which are able to integrate heterogeneous –omics datasets with high-throughput gene silencing experiments, such as those presented in Project Achilles^40^, will help us to gain further insights into the metabolic requirements that sustain growth in different cancer types. The use of both targeted and untargeted metabolomics approaches is particular important to identify novel metabolic phenotypes or key metabolites involved in different tumor stages.

Our GEA reveals MTAP as an essential gene. However, MTAP is frequently deleted in cancers concomitantly with CDKN2A locus^27^. This erroneous prediction could be explained for a compensatory benefit in losing MTAP expression. Indeed, it has been recently shown that MTAP deletion is synthetic lethal with inhibition of PRMT5^28^ suggesting that epigenetic modification could outcompete the detrimental consequences of MTAP loss in polyamine biosynthesis. Lack of integration of metabolic and signalling networks in our computational models is a limitation that requires further refinement.

In the same direction, the analysis of PNP and APRT indirectly exposes the need of improving existing genome-scale metabolic networks of human cells. A single annotation error led to an incorrect hypothesis (synthetic lethality of PNP and APRT), which was amended based on existing literature. This inconsistency was experimentally validated with gene silencing analysis of PNP and APRT in the ALL derived CEMO-1 cell line. In contrast to the information in the metabolic model considered, there is strong evidence suggesting that PNP does not consume adenine in human cells^39^. After correcting this mis-annotation, APRT turned out to be essential (Table 2), playing a key role in polyamine biosynthesis. We show here that iterative refinement of human metabolic networks must be driven by both computational and experimental work.

The results observed for the silencing of APRT in AML and ALL are remarkable. To our knowledge, the importance of APRT in cancer has not been explored. Instead, the APRT deficit has been found in a rare autosomal-recessive disorder that is compatible with life^41^ and results in renal damage^42^. This could open a therapeutic window for drug development. With the proof-of-concept here presented, we believe that further investigating the role of APRT in leukemias and other cancer types is promising and should be further considered in the future.

In particular, understanding and predicting the differential proliferative requirement of APRT for cancer cells is the next logical step. Our model predicts that the lack of APRT activity would result in excessive adenine. This metabolic scenario could have two molecular consequences leading to growth inhibition. On the one hand, adenine excess could be converted to DHA via xanthine dehydrogenase (XDH) (a phenomenon observed in individuals with APRT defects^42^ that is not considered in the metabolic model presented Folger *et al*.^5^), which in turn would hamper cancer cell proliferation (Supplementary Fig. 5). On the other hand, cells could reduce the flux of adenine production by MTAP in order to prevent the formation of DHA, and this would result in a reduction of polyamine production. In either case, lack of adenine clearance would be incompatible with tumor cell function.

Resistance to APRT knockdown could be achieved in the presence of alternative enzymes for adenine consumption or co-existing alterations in polyamine production genes. With respect to alternative enzymes for adenine consumption, different transporters could facilitate the excretion of accumulated adenine, including SLC29A1 and SLC29A2^43^, as well as SLC43A3^44^. However, we did not find statistically significant differences in our transcriptomic analysis between cells resistant and sensitive to APRT knockdown, thus suggesting that a compensatory effect in these cells is not at play. With regards to co-existing alterations in polyamine production genes, MTAP deletion, for example, would lead to a reduced production of adenine. In turn, this genetic alteration would uncouple polyamine flux from APRT activity and would be a determinant of sensitivity to APRT inhibition. Although cell lines considered in our study do not present MTAP deletion, we cannot discard that other alternative pathways may arise for the synthesis of polyamines. For all the above, further experimental demonstration of the metabolic consequences of APRT inhibition is warranted.

The differential gene expression profile between cancer cells sensitive or resistant to APRT silencing provided a molecular scenario that could contribute to clarify this cell-specific essentiality. On the one hand, lower expression of APRT in sensitive cell lines could imply that gene silencing results in a more profound depletion of the enzyme and hence a higher anti-proliferative effect (a *threshold* effect). On the other hand, the expression of SRM was upregulated in sensitive cell lines. This may indicate that the production of spermidine and adenine would be higher than in the resistant counterparts and, consequently, the pool of excessive adenine upon APRT inhibition would be increased. To reinforce this hypothesis, we computed the ratio of APRT/SRM (Fig. 4B). This ratio would reflect the production of adenine derived from polyamine synthesis that can be metabolized to AMP. A high ratio would imply that AMP is efficiently produced (due to high APRT or low production of adenine from polyamine synthesis), whereas a low ratio would imply that adenine is accumulated (due to the insufficient APRT activity to metabolize adenine produced from polyamines). Based on previous studies (mentioned above), adenine in excess would lead to the production of toxic DHA and growth suppression. In agreement with our data, cells sensitive to APRT-silencing present a low APRT/SRM ratio, suggesting that they are prone to DHA production and tumor suppression upon APRT perturbation. It is worth noting that due to the complexity of polyamine biosynthetic pathway, further enrichment of this pathway including cell-specific polyamine pool sizes and the rate of polyamine catabolism will provide a more refined view of gene essentiality^45^.

Our capacity to understand and go deeper into these issues will help us to rationally target polyamine production in different cancer types. The work presented here constitutes a step forward in this direction, opening an innovative way to target cancer cells through APRT inhibition. The use of integrative computational models in combination with heterogeneous and multidimensional molecular data is essential to address this challenge.

## Methods

### Constraint-based Modeling and Gene Essentiality Analysis

The core of Constraint-based Modeling (CBM) is the mass balance equation (Equation (1)). This equation represents the change in the concentration of a particular metabolite in terms of the number of molecules produced and consumed per unit of time. The concentration of a metabolite *c* (*c* = 1, …, *C*) is denoted as *x*_*c*_. For readers unfamiliar with metabolic modeling, for each reaction *r (r* = 1, …, *R)*, it is assigned a continuous flux variable *v*_*r*_ that represents its underlying activity. The total number of molecules produced (consumed) by each reaction is precisely its flux (*v*_*r*_) multiplied by the number of molecules produced (consumed) per unit of flux (*S*_*cr*_). *S*_*cr*_ (*c* = 1, …, *C*; *r* = 1, …, *R*) are the stoichiometric coefficients, which are grouped into the matrix *S*. As usual in the literature^46^, substrates have a negative stoichiometric coefficient, while products have a positive stoichiometric coefficient.

1 $$\sum _{r=1}^{R}{S}_{cr}\cdot {v}_{r}=\frac{d{x}_{c}}{dt},c=1,\mathrm{..}.,C$$

When we consider metabolic networks involving a large number of metabolites and reactions, the resulting set of differential equations cannot be solved due to lack of experimental information (concentration data and kinetic parameters) and its underlying complexity. Nevertheless, equation (1) can be simplified by assuming steady state, *i.e*. there is no effective variation in the concentration of considered metabolites over time. After forcing the steady-state assumption, equation (1) converts to equation (2), which is the main constraint used in CBM. In the analysis presented in the Results section, we used the stoichiometric matrix resulting from the cancer metabolic model presented in Folger *et al*.^5^.

2 $$\sum _{r=1}^{R}{S}_{cr}\cdot {v}_{r}=0,c=1,\mathrm{..}.,C$$

In addition, it is usual to have reversible reactions (*Rev*), *i.e*. reactions that can perform in both directions. In these cases their flux can be positive and negative depending if it performs in the forward and backward direction, respectively. On the other hand, if the reaction is defined as irreversible (*Irr*), it can only operate in the forward direction, therefore its activity must be greater or equal to zero. This is reflected in equation (3). Again, in the analysis presented in the Results section, we classified reactions into reversible/irreversible according to the cancer metabolic model presented in Folger *et al*.^5^.

3 $${v}_{r}\ge 0,\forall r\in Irr$$

It is also common the introduction of an upper (*u*_*r*_) and lower (*l*_*r*_) bound for metabolic fluxes, as observed in equation (4). As noted above, for irreversible reactions, *l*_*r*_ = 0. In case that the growth medium is known, we fixed to zero fluxes for intake reactions associated with non-available substrates. When no additional experimental information is available, *u*_*r*_ = *M* and for reversible reactions *l*_*r*_ = −*M*, where *M* is a large positive scalar. In the analysis presented in the Results section, we used the same growth medium and upper and lower bounds found in the cancer metabolic model in Folger *et al*.^5^.

4 $${u}_{r}\ge {v}_{r}\ge {l}_{r},r=1,\mathrm{..}.,R$$

The system of equations (2–4) is typically underdetermined and, therefore, we need to establish a biologically meaningful objective (optimization) function so as to find a particular solution. CBM allows us to identify the solution that maximizes the growth rate, subject to equations (2–4). To that end, an artificial reaction defining the metabolic requirements to produce new cells, in terms of building blocks and energy, is typically introduced. This artificial reaction is termed the biomass reaction and its flux represents the growth rate *v*_*bio*_, which is maximized:

5 $$\max \,({{\rm{v}}}_{{\rm{bio}}})$$

As noted in the Results section, we used the biomass reaction defined for a generic human cell found in Folger *et al*.^5^ and extended it with polyamines (spermidine, putrescine and spermine) in the amounts found in other reconstructions (see Supplementary Note 1).

Using equations (2–5), we can conduct Gene Essentiality Analysis, which aims to determine the set of essential and synthetic lethal genes. Essential genes are those whose individual deletion disables the organism/cell to support growth, while synthetic lethals refer to pairs of non-essential genes whose simultaneous deletion is lethal for growth. We describe below a systematic algorithm to determine essential genes.

Firstly, based on boolean rules that provide the relationship between genes and their encoded enzymes (proteins), available in the cancer metabolic model presented in Folger *et al*.^5^, we can find the subset of reactions inhibited when a particular gene *g* (*g* = 1, …, *G*) is not active, denoted as *N(g)*. This constraint is added into CBM (through equation (4)) and the maximum growth rate (*v*_*bio*_) is recalculated. If the maximum growth rate is below a certain arbitrary threshold, then such gene *g* is included in the list of essential genes. This arbitrary threshold represents the minimum biomass production to guarantee cellular proliferation. In the study presented in this manuscript, we considered the minimum biomass production rate to be 10^-4^ units, measured in grams per dry weight per hour (gDW/h). A sensitivity analysis for this threshold is included in Supplementary Note 1. This framework can be naturally extended so as to account for double gene deletion.

We used IBM Ilog Cplex in a Matlab environment to solve the underlying linear optimization problems defined by equations (2–5) upon different gene knockout perturbations. The computations were carried out on a 64 bit Intel Xeon E5-1620 v2 at 2.70 GHz (4 cores) and 16 GB of RAM.

### Cell culture, nucleofection and proliferation assay

Acute leukemia cell lines were maintained in culture in RPMI 1640 medium supplemented with fetal bovine serum at 37 °C in a humid atmosphere containing 5% CO_2_. All cell lines were tested for mycoplasma (MycoAlert Sample Kit, Cambrex) and no positive results were obtained. Gene silencing were done using a Nucleofector II device (Amaxa GmbH, Köln, Germany). Cells were nucleofected with Silencer Select siRNA s9655 for PNP, s1505 (APRT A), UGAGCUGGAGAUUCAGAAA (APRT B) and AGCUGGAGAUUCAGAAAGA (APRT C) for APRT and Silencer Select Negative Control-1 siRNA (Ambion, Austin, TX) at a final concentration of 50nM. Briefly, 1 × 10^6^ of cells were resuspended in 100 µL of supplemented culture medium with 50nM of APRT siRNAs or Silencer Select Negative Control-1 siRNA (Ambion, Austin, TX) and nucleofected with the Amaxa nucleofector apparatus using the following programs: G-009 (CEMO-1), C-005 (MY, MOLT-4,), C-013 (PEER) and V-001 (KG-1). Nucleofection was performed twice with a 24h interval. 48h after the second nucleofection, *PNP* and *APRT* mRNA expression was analyzed by Q-RT-PCR (*GUS* was employed as the reference gene). Nucleofection efficiency was determined by flow cytometry using the BLOCK IT Fluorescent Oligo (Invitrogen Life Technologies, Paisley, UK).

Cell proliferation was analyzed by MTS-based CellTiter 96® Aqueous Assay (Promega Corp., Madison, WI) that was used following the manufacturer’s instructions. Data were calculated as the percentage of total absorbance of APRT nucleofected cells/absorbance of control cells.

### Q-RT-PCR

Total mRNA was extracted with Trizol© Reagent 5791 (Life Technologies, Carlsbad, CA, USA) following the manufacturer’s instructions. RNA concentration was quantified using NanoDrop Specthophotometer (NanoDrop Technologies, USA). Reverse transcription was performed on 1 µg of total RNA, after heating at 70 °C for 5 min, with random hexamers as reaction primers. The reaction was carried out at 42 °C for 45 min in the presence SuperScript® II Reverse Transcriptase (Life Technologies, Carlsbad, CA, USA).

Real time PCR was performed in a 7300 Real Time System (Applied Biosystems, Foster City, CA), using 20ng of cDNA in 12 µl reaction volume with 0.25 µmol/l of each primer (APRT F: 5′-cactctgtgggcctcctatt-3′; APRT R: 5′-agatcatccacgacgaccac-3′; PNP F: 5′-catgctgatccgtgaccata-3′; PNP R: 5′-atcagacatggcagggaaac3′; GUS F: 5′-gaaaatatgtggttggagagctcatt-3′; GUS R: 5′-ccgagtgaagatccccttttta-3′), and SYBR® Green PCR Master Mix (Life Technologies, Carlsbad, CA, USA). Amplification of glucuronidase beta (*GUS*) transcript was performed as a reference gene using specific primers and TaqMan probe (Applied Biosystems, Foster City, CA). The relative expression of each gene was quantified by the Log 2^(−ΔΔCt)^ method using the gene *GUS* as an endogenous control.

### Gene expression data

We carried out a differential expression analysis between cells sensitive and resistant to APRT knockdown. In particular, we compared 3 samples of PEER (sensitive) and 3 samples of KG-1 (sensitive) against 5 samples of MOLT-4 (resistant). For the rest of cell lines considered in the main text, we could not find public microarrays data available. Microarrays data were obtained from Gene Expression Omnibus^47^. See Supplementary Table 2 for details of samples used in this analysis. We background corrected microarray data, applied quantile normalization and log2. For differential expression analysis between the two conditions, we used the *limma* package^48^ and selected those genes with an adjusted p-value < 0.05 using FDR approach.

## Electronic supplementary material

Supplementary Information
