# Supplementary material for: In-silico gene essentiality analysis of polyamine biosynthesis reveals APRT as a potential target in cancer
Source: Sci Rep. 2017 Oct 30;7:14358. doi: 10.1038/s41598-017-14067-8 (PMC5662602; doi:10.1038/s41598-017-14067-8)
Supplement: Supplementary file 1 — Supplementary Information [file 41598_2017_14067_MOESM1_ESM.docx]

Supplementary Information

**In-silico gene essentiality analysis of polyamine biosynthesis reveals APRT as a potential target in cancer**

Jon Pey^1,2,§^, Edurne San José-Eneriz^3,4,§^, María Carmen Ochoa^3,4^, Iñigo Apaolaza^1^, Pedro de Atauri^5^, Angel Rubio^1^, Xabier Cendoya^1^, Estíbaliz Miranda^3,4^, Leire Garate^3,4^, Marta Cascante^5^, Arkaitz Carracedo^4,6,7,8^, Xabier Agirre^3,4^, Felipe Prosper^3,4*^ and Francisco J. Planes^1,*^

**^1^** Bioinformatics Group, CEIT and TECNUN, University of Navarra, San Sebastian, 20018, Spain

**^2^** Mathematics for Life (M4L), San Sebastian, 20018, Spain

**^3^** Hemato-Oncology Division, IDISNA, Centro de Investigación Médica Aplicada (CIMA), University of Navarra, Pamplona, 31008, Spain

**^4^** CIBERONC

**^5^** Department of Biochemistry and Molecular Biology, University of Barcelona, Barcelona, 08028, Spain

**^6^** CIC bioGUNE, Bizkaia Technology Park, 801 Building, 48160 Derio, Spain

**^7^** Ikerbasque, Basque foundation for science, 48011 Bilbao, Spain

**^8^** Biochemistry and Molecular Biology Department, University of the Basque Country (UPV/EHU), P. O. Box 644, E-48080 Bilbao, Spain.

§ Both authors equally contributed to this work

* To whom correspondence should be addressed. Tel: 00-34-943212800; Fax: 00-34-943213076; Email: [fplanes@tecnun.es](mailto:fplanes@tecnun.es). Correspondence may also be addressed to Felipe Prosper. Email: fprosper@unav.es

**Supplementary Note 1. Sensitivity analysis of different parameters fixed in the gene essentiality analysis for polyamines production in cancer.** Here we conduct a sensitivity analysis on the arbitrary thresholds selected to (i) determine the coefficients of polyamines in the biomass equation (10^-3^ mmol/gDW for *spermidine*, *putrescine* and *spermine* in the main text) and (ii) define the minimum rate of biomass production to consider cellular growth viable (10^-4^ h^-1^ in the main text). For the sake of simplicity, the sensitivity analysis will be focused on the list of essential genes. Of course, the conclusions can be naturally extended to synthetic lethals.

Firstly, we generated 1000 random groups of biomass coefficients for *spermine*, *spermidine* and *putrescine*. These coefficients are bounded between **10^-6^ and 1**. Note that this interval is sufficiently large to ensure that any biologically meaningful biomass composition of these metabolites is included.

After recalculating gene essentiality analysis for each of the 1000 cases, the set of essential genes obtained is the same to the one presented in the main paper. Therefore, **the coefficients of *spermine***, ***spermidine*** and ***putrescine* in the biomass equation do not affect the resulting list of essential genes and, therefore, the solution presented in the main text is sufficiently robust**.

After analyzing the consistency of the method regarding the biomass composition of polyamines, we will focus on the minimum rate of biomass production to consider cellular growth viable (denoted here *t*), arbitrarily set to 10^-4^ h^-1^ in the main text. We analyze below the robustness of our results in function of this threshold *t*. In particular, we evaluate how different are the set of essential genes for different *t* values. Note here that a gene is classified as essential if the maximum rate of biomass production turns out to be below *t* once it is knocked out. In this study we consider the set of essential genes obtained with *t* = 10^-4^ h^-1^ as the gold-standard, which is precisely the set of essential genes presented in the main text.

A discrepancy in the set of essential genes for a particular *t* value might arise because:

- A gene is classified as essential whilst being declared non-essential in the gold standard;
- A gene turns out to be non-essential while being included in the set of essential genes in the gold standard.

This dichotomy could be conveniently summarized in a single parameter: the accuracy here denoted *acc_t_* and calculated in Equation (S1):

| $acc_{t}=\frac{TP_{t}+TN_{t}}{TP_{t}+FP_{t}+TN_{t}+FN_{t}}$ | (S1) |
| --- | --- |

where, for a particular *t* value,  *TP_t_* represents the number of true positives (number of genes correctly pointed out as essential), *TN_t_* represents the number of true negatives (number of genes correctly classified as non-essential), *FP_t_* represents number of false positives (genes resulting essential that are non-essential in the gold standard set)*. FN_t_* is the number of false negatives (genes classified as non-essential whilst being essential in the gold standard set). In essence, *acc_t_* provides a picture of the correctly classified genes, accounting for both essential and non-essential genes.

We analyze below the evolution of *acc_t_* for 5000 logarithmically distributed *t* values between 10^-20^ and 10^3^. For each *t* value, we calculated the list of essential genes for 1000 different polyamines compositions in the biomass equation considered above. The results are summarized Supplementary Figure 1-2.


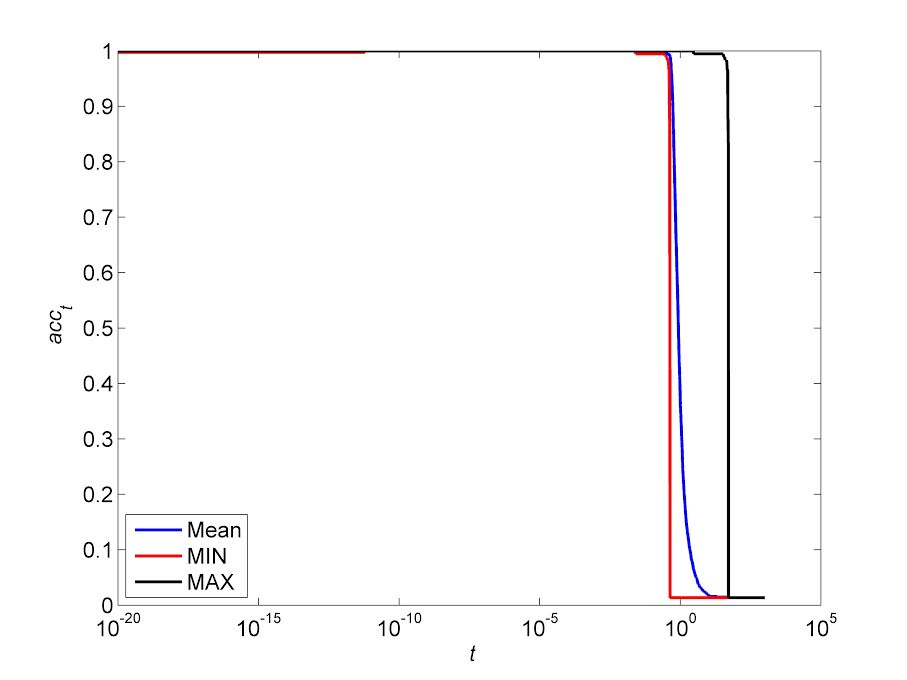


**Supplementary Figure 1: Accuracy** *acc_t_* **in predicting essential genes for polyamine biosynthesis for different *t* values**. Blue, red and black lines correspond to the mean, minimum and maximum value of *acc_t_* in the different simulated polyamines compositions in the biomass equation (1000 runs).

We can observe three different regions in Supplementary Figure 1: (i) a flat evolution from 10^-20^ to the vicinity of 1, (ii) a cliff starting close to 1 and (iii) a flat behavior reaching the end of the interval where *acc_t_* is equal to zero. To avoid the possible bias in the scale of Supplementary Figure 1, Supplementary Figure 2 zooms in on *acc_t_* values close to 1.


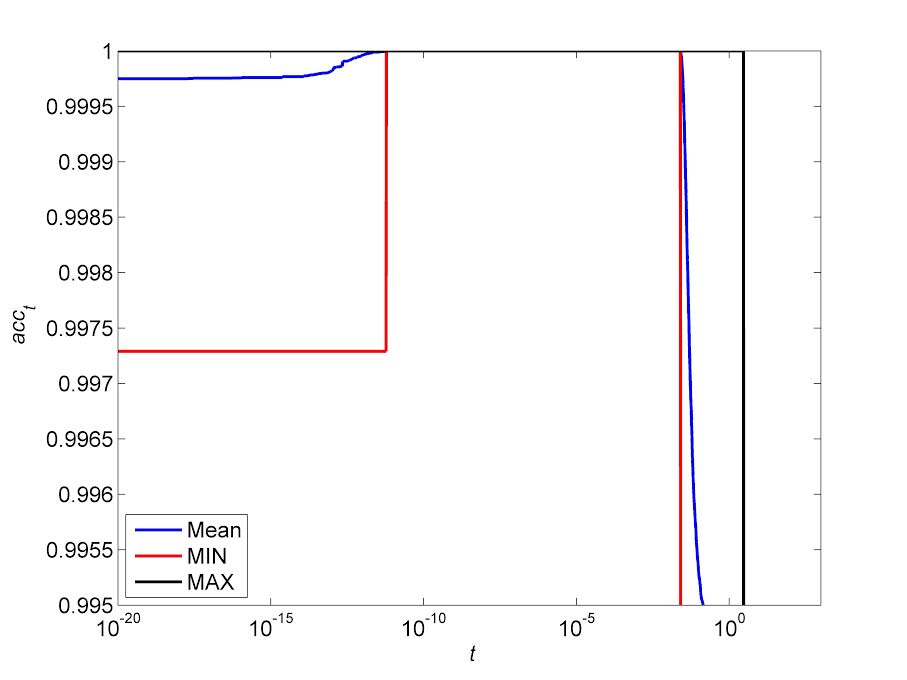


**Supplementary Figure 2: Zoom-in on** *acc_t_* **values in the interval [0.995, 1] in Figure S1.** Blue, red and black lines correspond to the mean, minimum and maximum value of *acc_t_* in the different simulated polyamines compositions in the biomass equation (1000 runs).

Supplementary Figure 2 clearly illustrates the presence of a new scenario for the smallest values of *t*. The accuracy in this region is slightly lower than 1 as a consequence of some essential genes in the gold standard being classified as non-essential (false negatives). When carrying out a more exhaustive analysis, we discovered that the knock-out of these misclassified genes resulted in a maximum biomass rate of 10^-11^. The exact value should be zero but numerical errors in the solving procedure lead to this discrepancy. For this reason the choice of *t* = 0 as the minimum rate to consider biomass production is not a good strategy to identify essential genes, as some of them may be incorrectly classified as non-essential due to numerical errors.

At this point, we showed that *t* = 0 is not a suitable threshold. For substantially high *t* values, conversely, some non-essential genes are misclassified as essential (false positives). As illustrated in Supplementary Figure 1-2, this inaccuracy is observed when t is close to 1. In this case, we incorrectly find as essential some genes that, when knocked out, do not disable the cellular system to sustain a biologically relevant amount of biomass but still below t.

Overall, based on the study presented here, we can conclude that:

- *t* = 10^-4^ is large enough to be robust against numerical error underlying the optimization process.
- *t* = 10^-4^ is small enough to be considered the minimum amount of biomass that the cell requires to sustain life. Note that the slowest growth rates of cancer cell lines reported in the literature (Yizhak et al, 2014) are in the order of 10^-2^.
- The coefficients of polyamines used in the biomass equation are sufficiently robust to ensure that do not affect the resulting set of essential genes.

.


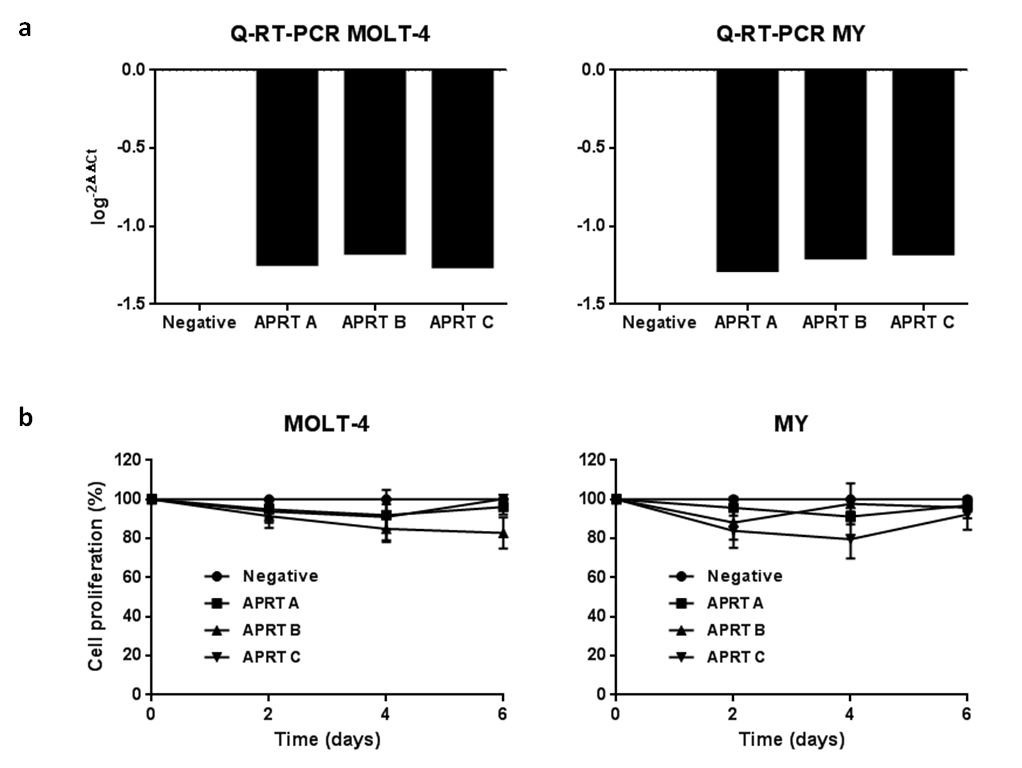


**Supplementary Figure 3: Gene silencing analysis of APRT in acute leukemias cell lines.** a) mRNA expression of *APRT* gene 48h after nucleofection with the siRNAs. Data are referred to GUS human gene and an experimental group nucleofected with Silencer Select Negative Control-1 siRNA. b) Cell proliferation of MOLT-4 and MY cell lines nucleofected with APRT siRNAs studied by MTS. Data represent mean ± standard deviation of three different experiments.


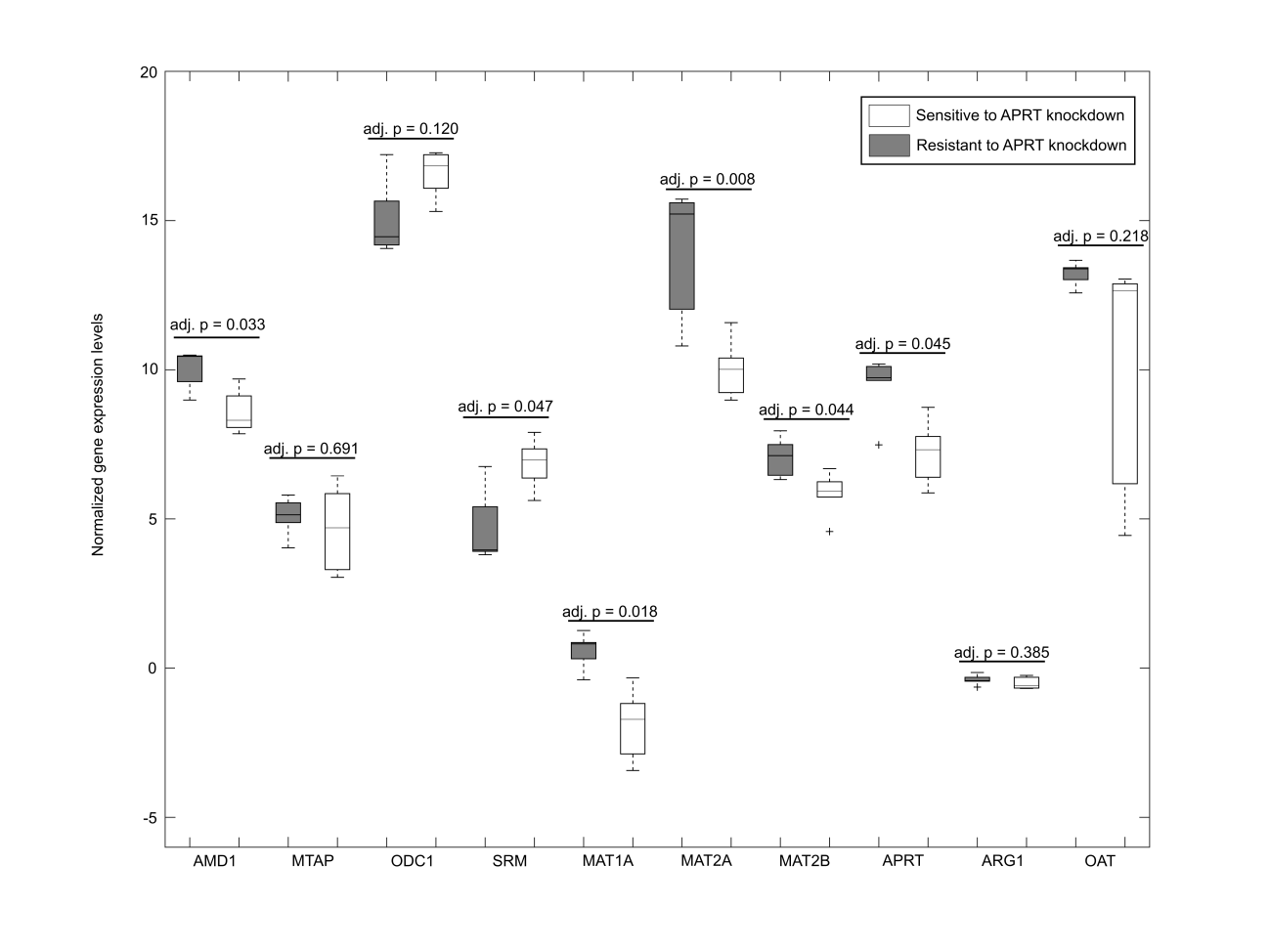


**Supplementary Figure 4. Differential expression analysis between cells sensitive and resistant to APRT knockdown for genes involved in the polyamines biosynthesis pathway.** Microarray data of 3 samples of KG1 (sensitive) and 3 samples of PEER (sensitive) was compared against 5 samples of MOLT-4 (resistant). See Methods section in the main text for further details. Note that MAT1A was not shown in Figure 4a because, as expected, it is not expressed in both conditions.


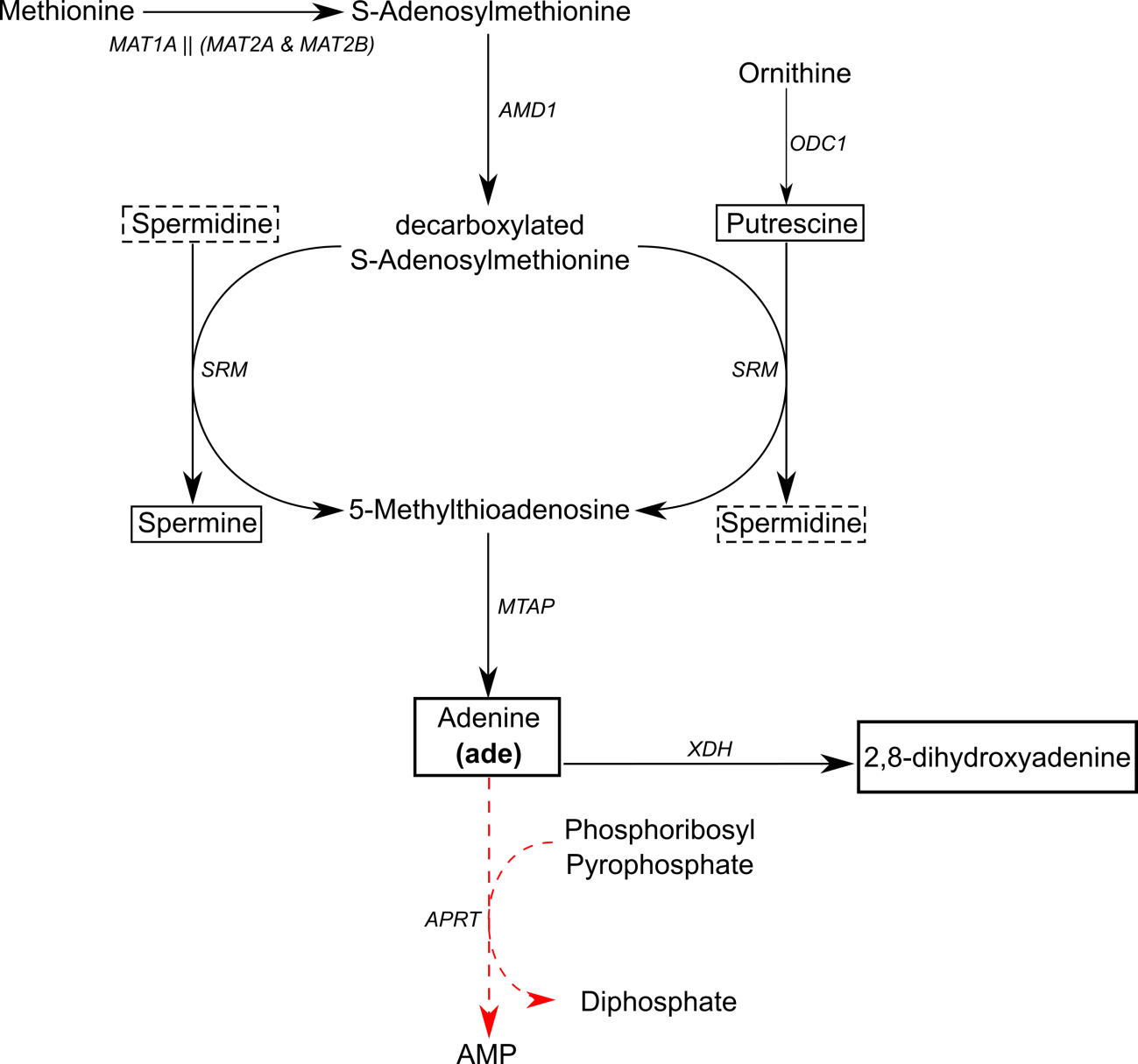


**Supplementary Figure 5. Hypothesis of metabolic adaptation of polyamine biosynthesis pathway upon APRT inhibition and adenine accumulation.** APRT (in red coloring) is deficient, which leads to adenine accumulation. Adenine is converted into 2,8-dihydroxyadenine (DHA) via XDH, an enzyme not considered in Folger et al, 2011.

**Supplementary Table 1: Reactions associated to APRT and PNP in the cancer metabolic model presented in Folger et al, 2011.**


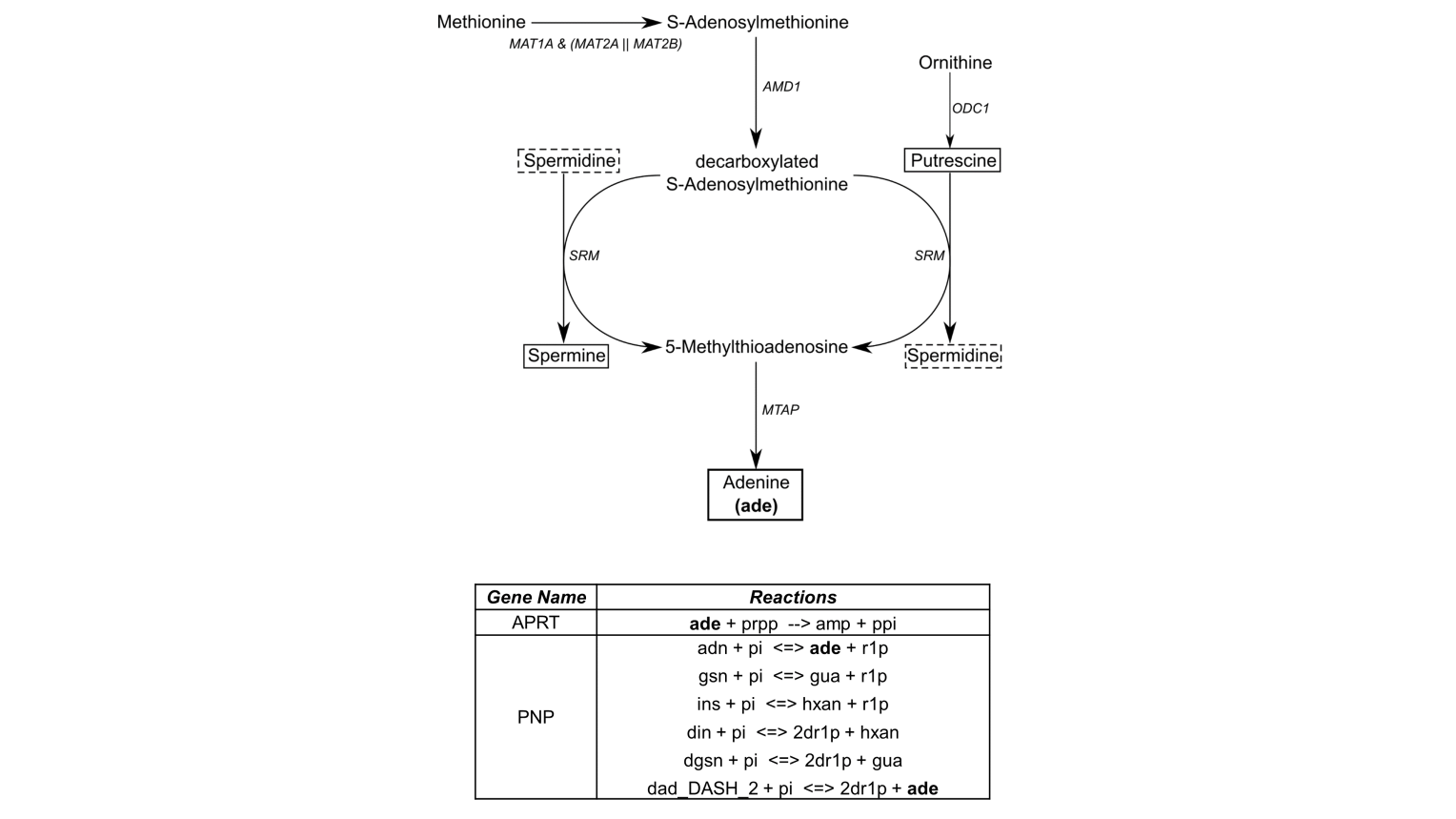


**Supplementary Table 2: List of GSMs involved in the expression analysis of cells sensitive and resistant to APRT knockdown.**

| **Cell Line** | **GSM** |
| --- | --- |
| MOLT-4 | GSM803617 |
| MOLT-4 | GSM1234408 |
| MOLT-4 | GSM1178423 |
| MOLT-4 | GSM1178424 |
| MOLT-4 | GSM1178425 |
| KG-1 | GSM887208 |
| KG-1 | GSM1446744 |
| KG-1 | GSM1251851 |
| PEER | GSM2399115 |
| PEER | GSM472169 |
| PEER | GSM887512 |

**References**

Folger, O. et al. Predicting selective drug targets in cancer through metabolic networks. Molecular systems biology 7, 501 (2011).

Yizhak, K. et al. Phenotype-based cell-specific metabolic modeling reveals metabolic liabilities of cancer. Elife, 3, e03641 (2014)
